# Management of Paediatric Acute Asthma in a Metropolitan Emergency Department: A Retrospective Audit

**DOI:** 10.1111/jpc.70446

**Published:** 2026-05-25

**Authors:** Yasmine Toufaili, Nounu Sarukkali

**Affiliations:** ^1^ The Children's Hospital at Westmead Sydney Children's Hospital Network Sydney Australia; ^2^ Fairfield Hospital Sydney Children's Hospital Network Sydney Australia; ^3^ The University of New South Wales Sydney Australia

**Keywords:** asthma, bronchodilator agents, child, emergency service, hospital, length of stay, quality improvement

## Abstract

**Aim:**

To evaluate adherence to the *Sydney Children's Hospital Network (SCHN) Asthma‐Acute Management Practice Guideline* in the management of paediatric patients presenting to a metropolitan emergency department (ED).

**Methods:**

A cross‐sectional retrospective audit of 153 paediatric ED presentations with acute asthma between March 2023 and February 2024 was conducted. Data extracted from electronic medical records included demographics, documented asthma severity, investigations, treatment, length of stay and discharge planning. Asthma severity was inferred retrospectively from documentation, which was often incomplete, limiting interpretation of patient management. Outcomes were compared with SCHN guideline recommendations. Data were analysed descriptively using means and proportions, with subgroup comparisons by admission status and severity classification.

**Results:**

Of 153 presentations, 56 (36.6%) resulted in admission. Documentation of key severity parameters was frequently incomplete, including speech (78%) and cyanosis (69%). Chest X‐rays were performed in 34.6% of cases. Mean bronchodilator burst duration exceeded guideline recommendations (salbutamol 46.7 min; ipratropium 47.7 min). Systemic corticosteroids were administered in 92% of patients, with appropriate dosing in 63%. Among 27 severe/life‐threatening cases, 8 (29.6%) received intravenous magnesium sulphate. Mean ED length of stay was longer in admitted patients than in discharged patients (6.5 vs. 4.3 h).

**Conclusions:**

Variation between local practice and SCHN guideline recommendations was identified in severity assessment, pharmacological management, imaging utilisation and discharge planning. Targeted quality improvement strategies, including implementation of ED short‐stay models, structured electronic severity assessment tools and staff education initiatives, may support improved adherence to guideline‐based care.

## Brief Points

- What is already known on this topic
  ○ Asthma is a common respiratory condition, affecting approximately 10% of Australian children.
  ○ Acute exacerbations of asthma vary in severity, ranging from mild to life‐threatening.
  ○ Optimal management of acute asthma should be guided by the severity of the exacerbation and evidence‐based clinical guidelines.
- What this paper adds
  ○ This audit describes variation in the local management of paediatric acute asthma compared with the SCHN guideline recommendations.
  ○ Observed ED LOS for both admitted and discharged patients was longer than the national benchmark of 4 h.
  ○ Potential strategies for quality improvement are suggested, including establishing an ED short‐stay unit, an electronic template for asthma severity, mandatory training modules for bronchodilator and corticosteroid delivery, asthma action packs, accreditation opportunities for criteria‐led discharges and focused teaching sessions.

## 1 Introduction

### 1.1 Background

Asthma is a common respiratory condition characterised by chronic airway inflammation, airflow obstruction and bronchial hyperresponsiveness [1]. It affects approximately 10% of Australian children and is the leading cause of disease burden among those aged 1–9 years, accounting for 2.5% of Australia's total disease burden in 2024 [2, 3]. In 2024–2025, there were 60 300 emergency department (ED) presentations for acute asthma in Australia, predominantly among children aged 0–14 years [3]. Exacerbations may be life‐threatening and require timely management to optimise patient outcomes. A New South Wales (NSW) cohort study found 5.4% of paediatric intensive care unit (PICU) admissions between 2010 and 2013 were children presenting with asthma [4].

### 1.2 Rationale

Clinical practice guidelines provide evidence‐based recommendations for the assessment and treatment of acute asthma. Previous audits have demonstrated inconsistent adherence to guideline recommendations in ED settings involving asthma severity assessment, bronchodilator administration, corticosteroid prescribing, treatment escalation and discharge planning [5, 6, 7]. This audit was undertaken to evaluate how current clinical practice aligns with the *Sydney Children's Hospital Network (SCHN) Asthma‐Acute Management Practice Guideline 2023*, as no formal ED asthma management guideline exists locally at Fairfield Hospital [8]. The 2023 SCHN guideline was selected as the audit standard because Fairfield Hospital operates within this network, making it the most relevant benchmark for evaluating guideline adherence in this setting.

### 1.3 Aims and Objectives

The primary aim of this audit was to assess adherence to key components of the *SCHN Asthma‐Acute Management Practice Guideline 2023* in paediatric ED presentations [8]. Secondary objectives were to evaluate documentation of asthma severity assessment, pharmacological management, investigation use and discharge practices to identify areas for quality improvement.

## 2 Methods

### 2.1 Design

This was a cross‐sectional retrospective audit conducted at the Fairfield Hospital ED, evaluating paediatric presentations of acute asthma from March 2023 to February 2024. The study was reported in accordance with the STROBE (Strengthening the Reporting of Observational Studies in Epidemiology) statement for observational studies [9].

### 2.2 Setting and Population

Inclusion criteria were as follows:

- Children aged 12 months to 15 years.
- ED diagnosis containing the term ‘asthma.’
- Patients who did not self‐discharge before medical review.

No additional screening of alternatively labelled respiratory presentations, such as ‘viral‐induced wheeze’, was undertaken. Children under 12 months were excluded due to diagnostic overlap with bronchiolitis [10]. The lower age limit of 12 months aligns with the SCHN and Royal Children's Hospital (RCH) definitions of paediatric asthma [8, 10].

### 2.3 Data Collection

Eligible cases were identified through the ED database using patient names and medical record numbers. Data were extracted from electronic medical records using a standardised data collection tool in Microsoft Excel and included demographics, clinical observations, medication administration records, investigations and discharge summaries. No additional patient contact was undertaken, and all data were de‐identified. Data extraction was performed by a single investigator, with a subset of cases independently reviewed and any discrepancies in severity assignment were resolved through consensus review against the SCHN criteria.

### 2.4 Audit Standards and Definitions

Audit standards were defined a priori using the *2023 SCHN Asthma‐Acute Management Practice Guideline*, which served as the benchmark for best practice [8]. Compliance was defined as adherence to guideline‐recommended assessment and management, and a compliance threshold of ≥ 80% was considered acceptable. The ≥ 80% threshold was selected a priori as a pragmatic benchmark, consistent with commonly used adherence cut‐offs reported in asthma literature, recognising that no universally accepted asthma‐specific threshold exists [11, 12].

Variables were selected to reflect key domains of guideline‐recommended care, including:

- Patient demographics (age, sex).
- Assessment of asthma severity (oximetry in air, heart rate, age‐appropriate ability to talk, wheeze intensity, accessory muscle use, altered consciousness and cyanosis).
- Treatment received (salbutamol and ipratropium bronchodilator burst therapy, prednisolone, intravenous (IV) magnesium sulphate).
- Investigations (chest X‐rays, pulmonary function tests [PFTs]).
- Length of stay (LOS).
- Discharge instructions (salbutamol weaning plan, asthma action plan [AAP]).

### 2.5 Data Analysis

**FIGURE 1:**
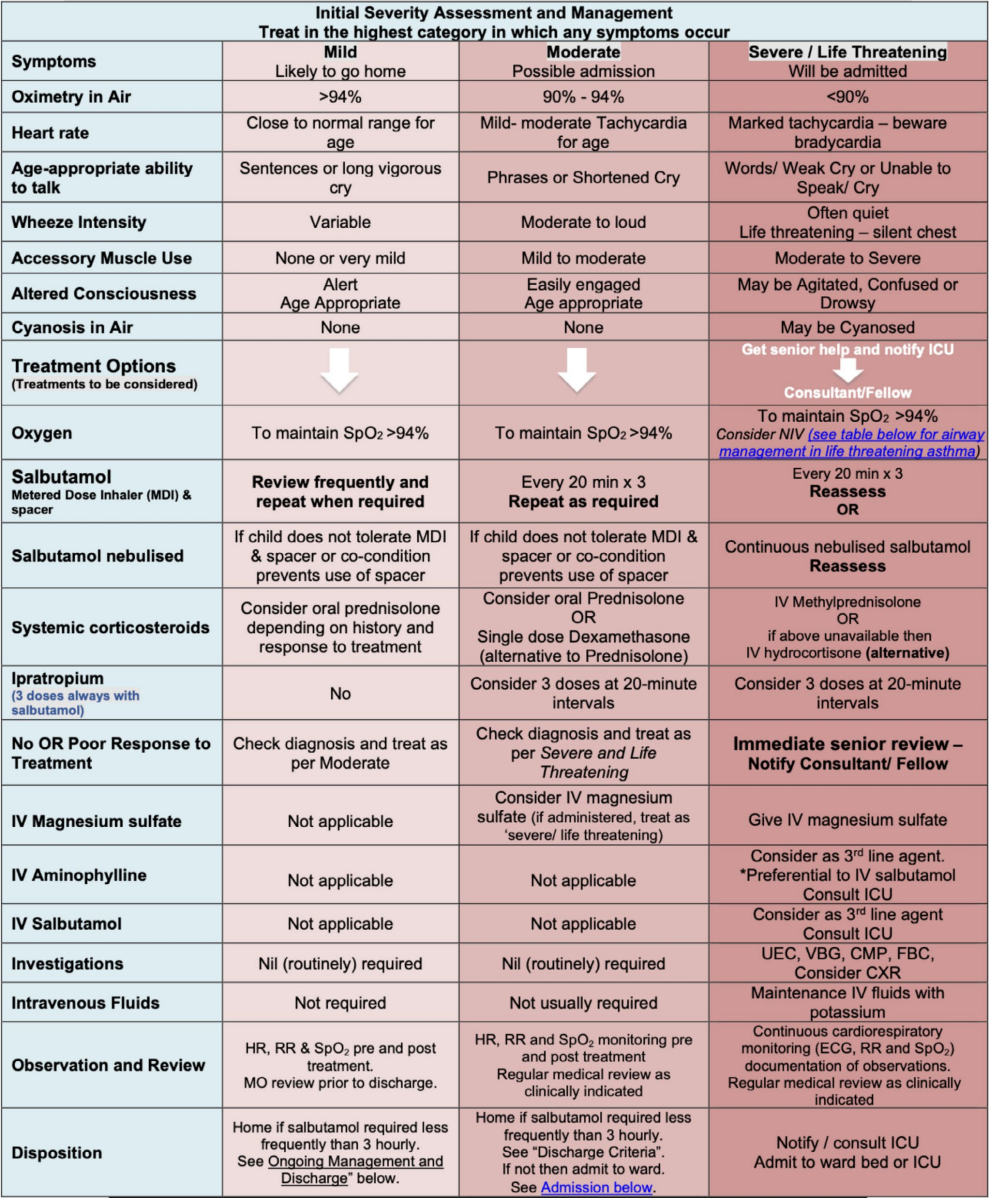
Initial assessment and management of acute asthma (8).

Data analysis was descriptive, with results presented as counts, proportions and measures of central tendency. As a quality assurance audit, the study was designed for descriptive evaluation, and therefore, inferential analysis was not performed. Analysis was performed using only available data, and the extent of missing variables is reported where relevant. Asthma severity was determined retrospectively using documented clinical parameters in accordance with SCHN criteria. Where documentation was incomplete or inconsistent, asthma severity was assigned using the most severe documented clinical feature available, based on the SCHN guideline approach to severity classification (Figure 1) [8]. Unrecorded variables were treated as missing rather than normal. Selected outcomes, including systemic corticosteroid and magnesium sulphate administration, were stratified by asthma severity.

### 2.6 Ethics Statement

The authors declare that the research presented adheres to the ethical principles outlined in the National Statement on Ethical Conduct in Human Research. All procedures were conducted in accordance with the ethical standards of the South Western Sydney Local Health District and the Declaration of Helsinki.

## 3 Results

### 3.1 Patient Characteristics

A total of 153 paediatric presentations of acute asthma were identified, of which 56 (36.6%) resulted in admission. The majority of patients were male (66.7%) (Table 1). Most presentations occurred in children aged 1–5 years (54.2%), followed by the 6–10 year age group (31.4%), and the 11–15 year age group (14.4%).

**TABLE 1:** Patient characteristics and asthma severity.

Month	Age (years)			Sex (*n*)		Severity (*n*)		
	1–5 (*n*)	6–10 (*n*)	11–15 (*n*)	Male	Female	Mild	Moderate	Severe/life‐threatening
March 2023	12	9	6	21	6	7	15	5
April 2023	8	6	1	9	6	6	7	2
May 2023	14	5	3	12	10	12	9	1
June 2023	9	1	2	7	5	3	4	5
July 2023	3	3	1	7	0	3	4	0
August 2023	4	7	0	6	5	5	4	2
September 2023	5	4	1	8	2	6	4	0
October 2023	8	0	1	8	1	3	4	2
November 2023	2	3	1	3	3	2	2	2
December 2023	7	2	1	6	4	3	6	1
January 2024	3	1	0	3	1	0	4	0
February 2024	8	7	5	11	9	7	6	7

### 3.2 Severity Assessment

Asthma severity was classified as mild in 57 cases, moderate in 69 and severe/life‐threatening in 27 (Table 1). Documentation of key severity indicators was frequently incomplete. Speech was not documented in 78% of cases and cyanosis in 69%, limiting the reliability of retrospective severity classification.

### 3.3 Investigations

No patients underwent PFT. Chest X‐rays were performed in 53 cases (34.6%). Reported findings included consolidation (*n* = 13), peribronchial thickening (*n* = 10), increased broncho‐vascular markings (*n* = 3), central bronchial cuffing (*n* = 6) and lung hyperinflation (*n* = 4).

### 3.4 Treatment

Mean bronchodilator burst duration exceeded guideline recommendations of 40 min (salbutamol 46.7 min; ipratropium 47.7 min). In 16% of cases, bronchodilator dosing did not align with guideline recommendations.

Systemic corticosteroids were administered in 92% of patients. Among these, 63% received doses aligned with age, weight and severity recommendations.

IV magnesium sulphate was administered to 13 patients. Among 27 severe/life‐threatening cases, 8 (29.6%) received magnesium sulphate.

### 3.5 LOS

Mean ED LOS was 4.3 h for discharged patients and 6.5 h for admitted patients (Table 2). Among the 56 admitted patients, the mean ward LOS was 1.3 days (Table 2).

**TABLE 2:** LOS.

Month	LOS in ED for discharged patients (h)	LOS in ED for admitted patients (h)	Number of paediatric ward admissions (*n*)	Average duration of paediatric ward admissions (days)
March 2023	3.70	4.82	8	0.9
April 2023	4.90	7.53	7	0.7
May 2023	3.23	7.85	7	1.5
June 2023	4.58	5.40	9	1.2
July 2023	5.35	6.62	2	1.5
August 2023	5.20	8.60	2	2
September 2023	4.23	6.95	1	1
October 2023	4.35	5.33	4	1.25
November 2023	4.03	4.62	2	2.5
December 2023	3.98	7.82	3	0.7
January 2024	4.20	6.80	2	1
February 2024	4.18	6.17	9	1.3

### 3.6 Discharges

AAPs were provided in 33% of cases, while 73% received a salbutamol weaning plan (Table 3). Two of the 56 admitted patients underwent criteria‐led discharge (CLD).

**TABLE 3:** Discharges.

Month	Criteria‐led discharge (*n*)		AAP provided (*n*)	Salbutamol weaning plan provided (*n*)
	Yes	No		
March 2023	0	8	5	20
April 2023	1	6	7	12
May 2023	1	7	7	15
June 2023	0	9	4	8
July 2023	0	2	3	4
August 2023	0	2	3	8
September 2023	0	1	1	8
October 2023	0	4	6	7
November 2023	0	2	2	4
December 2023	0	3	4	7
January 2024	0	2	0	4
February 2024	0	9	9	15

## 4 Discussion

This audit examined paediatric acute asthma management in a metropolitan ED over 12 months and identified clinically important variation between current ED practice and SCHN guideline recommendations [8]. While a ≥ 80% threshold was used to define acceptable adherence, consistent gaps were observed in severity assessment, pharmacological management, imaging utilisation and discharge planning. These findings suggest suboptimal adherence across domains, regardless of reasonable variation in the threshold applied.

### 4.1 Asthma Severity Assessment

Incomplete documentation of asthma severity parameters, particularly speech and cyanosis, was a prominent finding. Accurate severity assessments are essential for guiding treatment escalation and should be performed and documented systematically. Barriers to adherence may include clinician time constraints, absence of standardised referral pathways, long waiting lists and limited resources [13]. Implementation of a structured electronic medical record template incorporating all severity criteria could support more consistent documentation and clinical assessment.

### 4.2 LOS

ED LOS exceeded the 4‐h National Emergency Access Target for both discharged and admitted patients [14]. While system‐level factors were not formally assessed, prolonged observation periods required for bronchodilator therapy may be associated with extended LOS. Given the relatively short inpatient LOS (mean 1.3 days), some patients may be suitable for management in an ED short‐stay unit. Evidence indicates that these models may improve patient flow, patient satisfaction, cost‐effectiveness and hospital LOS [15].

### 4.3 Diagnostic Investigations

The use of chest radiography in one‐third of cases suggests potential overuse relative to guideline recommendations, which reserve imaging for specific clinical indications such as focal signs or suspected complications [8]. Although this audit did not assess clinical indications for imaging, higher imaging rates may be associated with false diagnoses of pneumonia and subsequent antibiotic overuse [16]. In a retrospective study of 643 children with acute asthma, 88% underwent chest radiography and radiographic abnormalities were associated with increased antibiotic prescribing [16]. Targeted clinician education may support more judicious imaging and support antibiotic stewardship.

PFT was not performed, reflecting consistency with guideline recommendations [8]. Although PFT can support diagnosis, normal results do not exclude asthma, limiting its utility in the acute setting.

### 4.4 Pharmacological Treatment

#### 4.4.1 Bronchodilator Therapy

Short‐acting beta‐2‐adrenergic receptor agonists (SABAs), such as salbutamol, are first‐line therapy for acute asthma exacerbations, with delivery via a metered‐dose inhaler (MDI) and spacer recommended for most patients [8]. Evidence demonstrates no superiority of nebulisers over MDIs in mild‐to‐moderate asthma [17]. Combining a short‐acting muscarinic acetylcholine receptor antagonist (SAMA), such as ipratropium, has been shown to reduce hospitalisation and improve lung function in moderate‐to‐severe cases [18].

In this cohort, the mean bronchodilator burst duration exceeded the guideline‐recommended 40 min (Table 4). In 16% of cases, the administered bronchodilator doses did not meet guideline recommendations. Although patient outcomes were not evaluated, this finding represents a deviation from recommended management and may reflect variability in care delivery.

**TABLE 4:** Summary of asthma management.

Treatment							
Duration of burst therapy (min)			Number that did not receive correct burst therapy (*n*)		Number received steroids (*n*)	Number of appropriate steroid administration (*n*)	Number that received IV magnesium sulphate (*n*)
Month	Salbutamol	Ipratropium	Salbutamol	Ipratropium			
March 2023	43	42	4	1	27	16	1 (severe/life‐threatening)
April 2023	46	40	2	2	15	10	1 (moderate)
May 2023	40	35	2	3	18	8	2 (both moderate)
June 2023	72	84	0	3	12	7	2 (severe/life‐threatening, mild)
July 2023	41	46	1	2	17	4	1 (mild)
August 2023	41	38	1	2	11	6	2 (both severe/life‐threatening)
September 2023	44	44	0	0	8	5	0
October 2023	42	45	0	0	8	6	0
November 2023	51	47	0	0	5	3	1 (severe/life‐threatening)
December 2023	45	49	0	1	9	7	0
January 2024	47	55	0	0	4	4	0
February 2024	48	47	0	0	16	11	3 (all severe/life‐threatening)

Potential contributing factors include high patient volume, variability in ED staff experience and the absence of a dedicated paediatric area. A 2025 cross‐sectional study found that only 4.4% of healthcare professionals had correct inhaler technique knowledge [19]. Establishing a structured ED short‐stay unit to support bronchodilator monitoring, alongside mandatory training on bronchodilator administration, may enhance clinical practice.

#### 4.4.2 Systemic Corticosteroids

Systemic corticosteroids are recommended in most asthma exacerbations and are associated with reduced hospital admissions and relapse rates [8, 20]. Evidence supports the use of single‐dose dexamethasone as a non‐inferior alternative to multi‐day prednisolone in mild‐to‐moderate cases, with the advantages of improved adherence and reduced vomiting [8, 20]. Awareness of this option may be enhanced through targeted clinician education sessions.

In our cohort, corticosteroids were administered in the majority of patients; however, only 63% received guideline‐recommended doses based on age, weight and asthma severity. This highlights an opportunity to improve prescribing accuracy, potentially through mandatory training modules.

#### 4.4.3 Magnesium Sulphate

IV magnesium sulphate is recommended for severe/life‐threatening asthma and may be considered in moderate cases unresponsive to bronchodilators [8]. A 2024 systematic review and meta‐analysis demonstrated an 85% reduction in hospitalisation and improved peak expiratory flow with magnesium sulphate use [21].

In this cohort, fewer than one‐third of severe/life‐threatening cases received magnesium sulphate, indicating a gap in escalation of care relative to guideline recommendations (Table 4). Although patient outcomes were not assessed, this finding may be associated with a higher risk of clinical deterioration and prolonged hospital stay [21]. Review of severe/life‐threatening cases in morbidity and mortality meetings may support appropriate magnesium sulphate use and adherence to the SCHN guideline.

### 4.5 Discharge Planning

#### 4.5.1 AAPs

Only one‐third of patients received an AAP, which is lower than the rate of 67.2% reported by the Australian Bureau of Statistics in 2022 [22]. This represents a key gap in care, as AAPs are associated with reduced asthma‐related mortality and unplanned healthcare visits [23, 24].

Barriers to providing AAPs in the ED setting may include limited access to standardised resources and time constraints [25]. Implementation of an easily accessible ED asthma discharge pack incorporating both an AAP and a salbutamol weaning plan may improve uptake [25].

#### 4.5.2 CLDs

CLDs enable nurse‐initiated patient discharge once predefined criteria are met. Evidence from the British Medical Journal indicates that CLDs in paediatric asthma reduce LOS without increased hospital re‐presentation [26]. Only 2 of the 56 admitted patients in this audit were discharged via CLD, indicating limited utilisation. Expanding nursing staff CLD accreditation may facilitate increased implementation.

## 5 Limitations

This study is limited by its retrospective design and reliance on clinician documentation, which may introduce misclassification bias, particularly in severity assessment. Incomplete documentation of severity assessments may affect the reliability of severity‐based analyses and downstream conclusions, potentially attenuating observed differences in management between asthma categories. Additionally, variables used to classify asthma severity were evaluated as audit outcomes, introducing the potential for circularity.

The inclusion criterion requiring an ED diagnosis containing the term ‘asthma’ may have excluded patients labelled with alternative diagnoses, such as ‘viral‐induced wheeze’. This is particularly relevant in children under 6 years of age, where there is diagnostic overlap between asthma and viral‐induced wheeze. As broader wheeze‐coded presentations were not reviewed, the number of missed potentially eligible cases is unknown. This introduces potential selection bias and limits the representativeness of the sample, as findings may underestimate the burden of asthma presentations in this ED and do not capture management patterns among these excluded cases.

The relationship between imaging findings and antibiotic prescribing was not assessed, limiting the interpretation of the downstream clinical impact of imaging practices. Additionally, corticosteroid dosing errors were analysed in aggregate, limiting detailed evaluation of error patterns across age and severity groups.

## 6 Conclusion

This audit identified clinically meaningful gaps between current practice and guideline‐recommended care in paediatric acute asthma management. Targeted interventions—including establishment of an ED short‐stay unit, structured documentation tools, clinician education and standardised discharge resources—may improve guideline adherence and optimise patient outcomes. Prospective quality improvement initiatives incorporating robust statistical analysis are warranted to evaluate the impact of such interventions in paediatric asthma management.

## Funding

The authors have nothing to report.

## Ethics Statement

Ethical approval was obtained by the South Western Sydney Local Health District Human Research Ethics Committee via the low or negligible risk review pathway, and the site specific assessment was authorised.

## Consent

A waiver of consent was approved in the ethical approval process.

## Conflicts of Interest

The authors declare no conflicts of interest.

## Supporting information


**Data S1:** STROBE statement—Checklist of items that should be included in reports of *cross‐sectional studies*.

## Data Availability

The data that support the findings of this study are available from the corresponding author upon reasonable request.
